# Molecular Dynamics Simulation Reveal the Structure–Activity Relationships of Kainoid Synthases

**DOI:** 10.3390/md22070326

**Published:** 2024-07-22

**Authors:** Zeyu Fan, Xinhao Li, Ruoyu Jiang, Jinqian Li, Fangyu Cao, Mingjuan Sun, Lianghua Wang

**Affiliations:** Department of Biochemistry and Molecular Biology, College of Basic Medical Sciences, Naval Medical University, Shanghai 200433, China; zeyufan@smmu.edu.cn (Z.F.);

**Keywords:** kainoid synthases, structure–activity relationships, molecular dynamics simulation, promiscuity

## Abstract

Kainoid synthases are key enzymes in the biosynthesis of kainoids. Kainoids, as represented by DA and KA, are a class of naturally occurring non-protein amino acids with strong neurotransmitter activity in the mammalian central nervous system. Marine algae kainoid synthases include PnDabC from diatoms, which synthesizes domoic acid (DA), and DsKabC and GfKabC from red algae, which synthesize kainic acid (KA). Elucidation of the catalytic mechanism of kainoid synthases is of great significance for the rational design of better biocatalysts to promote the industrial production of kainoids for use in new drugs. Through modeling, molecular docking, and molecular dynamics simulations, we investigated the conformational dynamics of kainoid synthases. We found that the kainoid synthase complexes showed different stability in the simulation, and the binding and catalytic processes showed significant conformational transformations of kainoid synthase. The residues involved in specific interactions with the substrate contributed to the binding energy throughout the simulation process. Binding energy, the relaxed active pocket, electrostatic potential energy of the active pocket, the number and rotation of aromatic residues interacting with substrates during catalysis, and the number and frequency of hydrogen bonds between the individual functional groups revealed the structure–activity relationships and affected the degree of promiscuity of kainoid synthases. Our research enriches the understanding of the conformational dynamics of kainoid synthases and has potential guiding significance for their rational design.

## 1. Introduction

**Figure 1:**
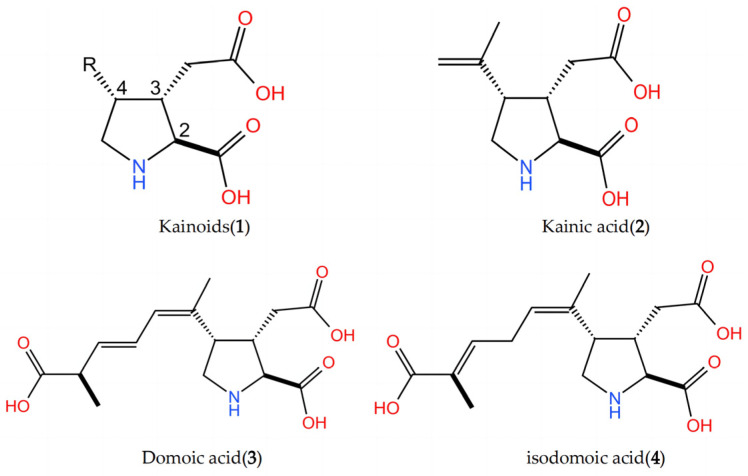
Structures of kainoids **1**–**4**.

The kainoids (**1**) are 2,3,4-trisubstituted pyrrolidine derivatives that contain a glutamic acid moiety and various substituents at the four-position of the pyrrolidine ring (Figure 1). Domoic acid (DA) (**3**) [1] and kainic acid (KA) (**2**) [2], the representatives of the kainoids, were first isolated from Japanese marine algae *Chondria armata* and *Digenea simplex*. Both types of seaweed are used as insect repellents in traditional Chinese and Japanese medicine. Later, it was found that DA can also be synthesized in some diatoms, such as *Pseudonitzschia* and *Nitzschia* [3], while KA can be synthesized in some red algae, like *Grateloupia filicina*. DA and KA act as excitatory amino acid receptor agonists, and KA is widely used as a tool in neuropharmacology to stimulate nerve cells and mimic disease states, such as epilepsy [4], Alzheimer’s disease, and Huntington’s chorea [5]. As members of a novel class of marine meroterpenoids, the basic medical research application and biosynthetic pathway of DA and KA have attracted considerable attention in recent years [6,7,8].

DA and KA are currently synthesized by chemical or biological techniques. KA can be successfully synthesized by constructing 2,3,4-tri-substituted pyrrolidine scaffolds through SMI2-promoted stereocomplementary ring closure reactions [9]. The key structure of the C1′-C2′ *Z*-configuration of DA is completed using the unsaturated lactam structure. Three continuous stereocenters on the pyrrolidine ring are effectively established through a sequential rearrangement reaction, and side chain fragments are introduced by modifying the Julia–Kocienski reaction [10]. Compared with chemical synthesis, biosynthesis frequently shows higher regional and stereoselectivity. In the DA biosynthetic pathway, isopentenyltransferase (IPT) uses geranyl pyrophosphate (GPP) and L-Glu as substrates to synthesize L-NGG. Then, 7′-carboxy-L-NGG (cNGG) is synthesized by the cytochrome P450 enzyme. Finally, isodomoic acid is synthesized by kainoid synthases PnDabC and then converted into domoic acid [6,7]. In the KA biosynthetic pathway, IPT uses dimethylallyl pyrophosphate (DMAPP) and L-Glu as substrates to synthesize prekainic acid (PKA). Then, KA is synthesized by kainoid synthases DsKabC and GfKabC. Kainoid synthases have been found to have catalytic promiscuity [8], among which, GfKabC has been found to have higher catalytic promiscuity. Understanding the relationship between the structure, dynamics, and function of kainoid synthases is essential for the rational design and effective control of DA and KA biosynthesis.

In this study, we used structural prediction, molecular docking, and molecular dynamics simulations (MDS) to investigate the conformational dynamics of kainoid synthases. Firstly, AlphaFold2 was used to predict the tertiary structures of three kainoid synthases: PnDabC, DsKabC, and GfKabC. Molecular docking was then performed using AutoDock. Secondly, the MDS over 500 ns showed that there were binding and catalytic processes in the entire simulation. The conformational transformations of key residues of kainoid synthases played important roles in the binding and catalytic processes. Finally, we found that the relaxation degree of the active pocket, the number and rotation of aromatic residues interacting with substrates during catalysis, the number of potential π-cation bonds, and the differences in binding energy may contribute to the promiscuity of kainoid synthases.

## 2. Results and Discussion

### 2.1. AlphaFold2 Predicts the Kainoid Synthases Structure

**Figure 2:**
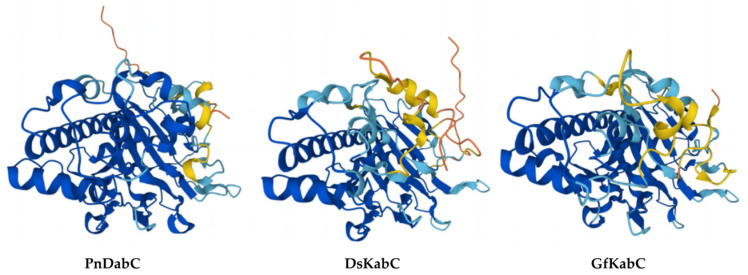
Kainoid synthases structure predicted by AlphaFold2.

**Figure 3:**
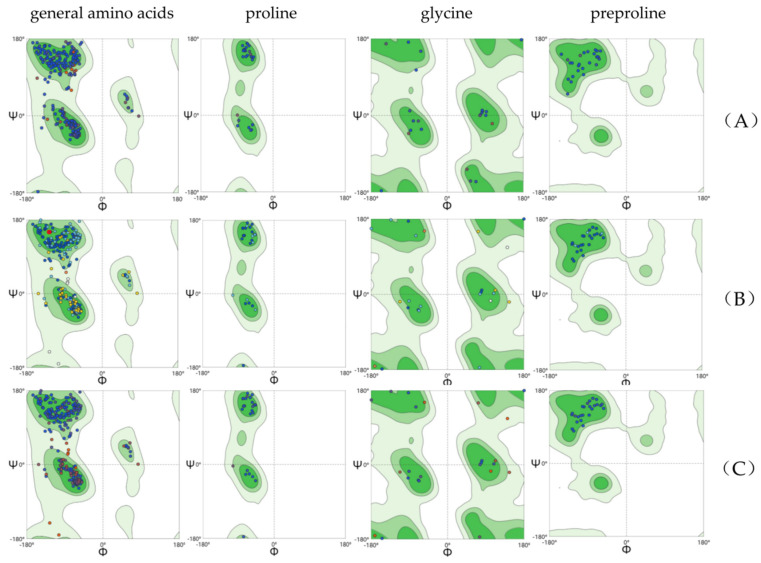
Ramachandran plots of kainoid synthases. From left to right are Ramachandran plots of general amino acids, proline, glycine, and preproline. (**A**) Ramachandran plots of PnDabC; (**B**) Ramachandran plots of DsKabC; (**C**) Ramachandran plots of GfKabC.

AlphaFold2 was used to predict the tertiary structures of kainoid synthases (Figure 2). The pLDDT values of the three kainoid synthases were 89.44, 88.03, and 87.75, respectively. The Ramachandran plots explain the relationship between ϕ and ψ of the two dihedral angles of the residue in a protein. In an amino acid, ϕ refers to the dihedral angle between Cα and N, and ψ refers to the dihedral angle between Cα and carboxyl C. The Ramachandran plot is divided into four regions: most favored, additional allowed, generously allowed, and disallowed, with darker regions indicating greater suitability. For a high-quality model structure, 90% of the ϕ and ψ values should be in the darkest area. The Ramachandran favored (%) values of PnDabC, DsKabC, and GfKabC were 96.23%, 94.75%, and 94.75%, respectively. SWISS-MODEL was used to evaluate the structures predicted by AlphaFold2, and it was found that the structures of PnDabC, DsKabC, and GfKabC all conformed to stereochemical conformation, and these structures could be used for further analysis (Figure 3). We used AutoDock to perform molecular docking. After that, a 500 ns molecular dynamics simulation was performed.

### 2.2. Overall Stability of the Kainoid Synthase–Substrates Complex

**Figure 4:**
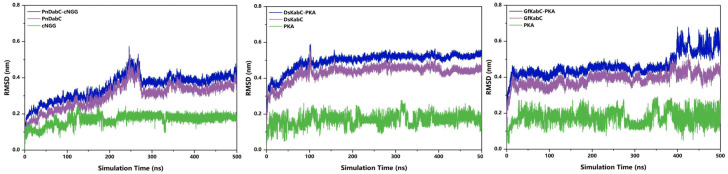
RMSD of kainoid synthases–substrates, kainoid synthases, and substrates.

**Figure 5:**
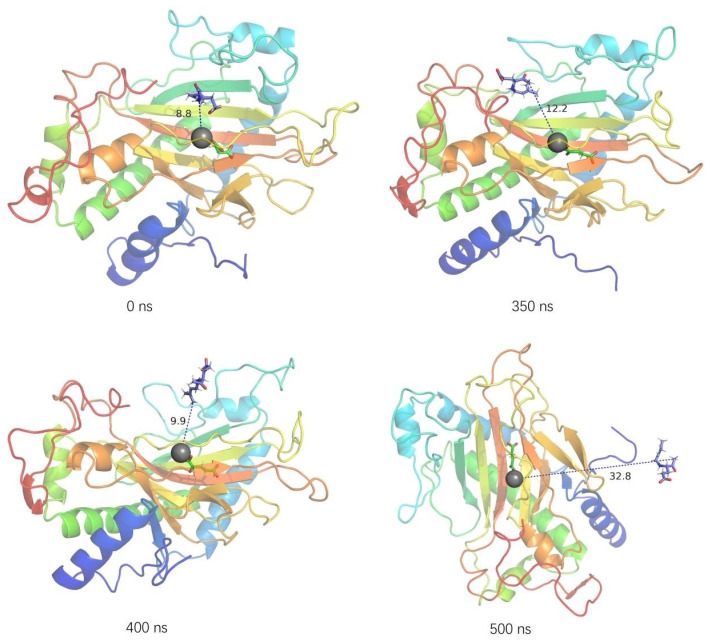
The conformational change of GfKabC-PKA (the purple stick–ball model is PKA, the black ball model is Fe^2+^, the purple dash is the interaction between the GfKabC and PKA, GfKabC coloring style for rainbow).

Root mean square deviation (RMSD) represents the sum of atomic deviations between all conformations and target conformations at a certain time. It is an important basis for measuring system stability. In the substrate-bound systems, the RMSD values of PnDabC and DsKabC converged at around 0.40 nm and 0.53 nm, respectively, indicating that the complex structures were gradually stabilizing (Figure 4). For the first 350 ns, the RMSD value of the GfkabC-PKA complex rose rapidly and converged to 0.45 nm, and finally experienced a large fluctuation during 350–500 ns. The RMSD of GfKabC was not synchronized with the complex after 350 ns, indicating that the RMSD fluctuation of the complex came from the conformational change of PKA binding to GfKabC. The trajectory of PKA binding to GfkabC in the complex was analyzed (Figure 5). At the time points 0, 350, 400, and 500 ns, the distance between PKA and the Fe atom increased from the initial 8.8 nm to the final 32.8 nm. This indicates that PKA gradually separated from the initial binding site after 350 ns.

**Figure 6:**
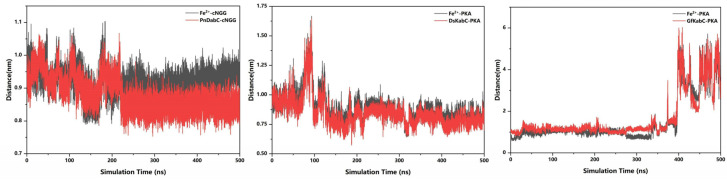
Distance between the substrate and kainoid synthase.

In order to analyze the state of the substrate on the surface of the kainoid synthase and obtain the initial docking site of the substrate, we analyzed the distance between the center of mass (Fe^2+^) of the initial docking site and the center of the substrate, as well as the distance between the substrate and the center of mass of the kainoid synthase. These distances reflect the binding state of the substrate to the kainoid synthase. The distance between cNGG and PnDabC, and the distance between cNGG and Fe^2+^, gradually stabilized, indicating that the binding between cNGG and PnDabC was gradually stabilizing (Figure 6). Although the distance between PKA and Fe^2+^ fluctuated within the first 100 ns, the fluctuation value did not exceed 1 nm. The distance between the PKA and DsKabC center and Fe^2+^ gradually stabilized, indicating that the combination of PKA and DsKabC was gradually stabilizing. However, the distance between PKA and GfKabC, and the distance between PKA and Fe^2+^, remained stable within the first 350 ns, indicating that the binding of PKA and GfKabC was relatively stable during this period. After 350 ns, these distance values gradually showed relatively drastic fluctuations, indicating that the binding between PKA and GfKabC was no longer stable.

**Figure 7:**
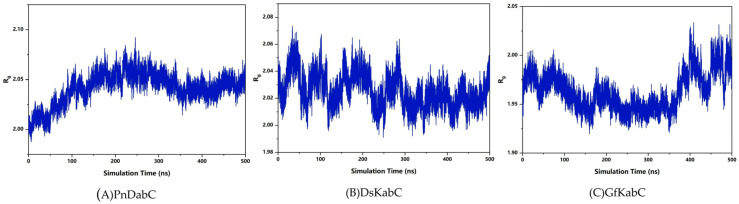
Radius of gyration of the kainoid synthases in simulation.

**Figure 8:**
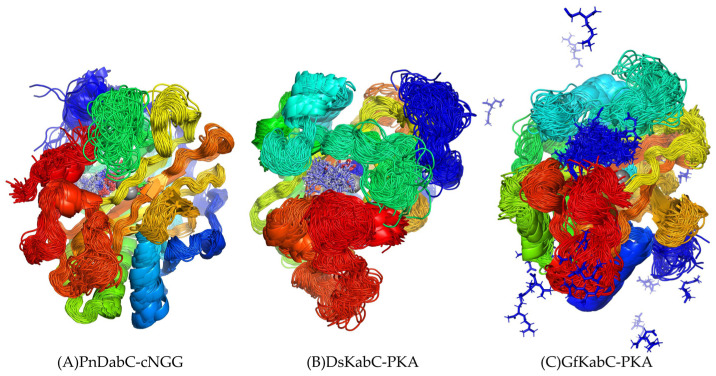
Superposition of the kainoid synthases and substrates in simulation.

The radius of gyration (Rg) can be used to characterize the tightness of the complex structure. According to the analysis, the Rg of the PnDabC-cNGG complex rose slowly and then remained stable, indicating that the overall structure of the complex was gradually stabilizing. The Rg fluctuation amplitude of the DsKabC-PKA complex decreased and stabilized gradually. For the GfKabC-PKA complex, the Rg gradually decreased over the first 350 ns, then suddenly increased and experienced sharp fluctuations after 350 ns, indicating that the overall structure of the GfKabC-PKA complex remained stable in the 0–350 ns range (Figure 7). In the range of 350–500 ns, the overall Rg increased and fluctuated as PKA departed from the initial binding site of GfKabC. Superposition of the substrate with kainoid synthases revealed that cNGG in PnDabC and PKA in DsKabC had a high degree of overlap, meaning the substrate was distributed at the initial binding site, indicating the binding of cNGG with PnDabC and PKA with DsKabC was stable. In GfKabC, PKA was distributed in other locations on the surface of GfKabC, indicating that there were both stable and unstable periods in the combination of PKA and GfKabC (Figure 8).

### 2.3. Structural Flexibility of Kainoid Synthases

**Figure 9:**
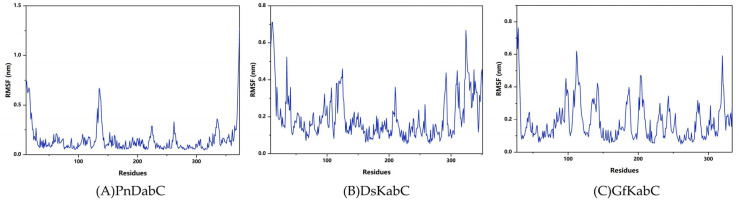
RMSF of kainoid synthases.

**Figure 10:**
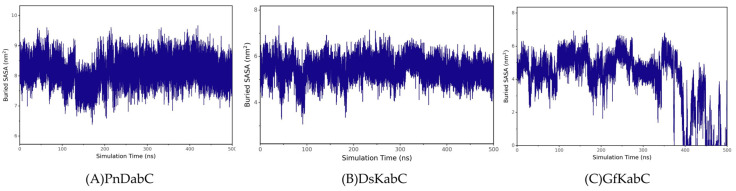
Buried SASA of kainoid synthases.

In order to explore the structural flexibility of kainoid synthases, root-mean-square fluctuations (RMSF) of the substrate-free state were calculated. In the substrate-free state, the flexible regions of PnDabC included the 122G-145V loop, 218P-231R loop, 258V-266V loop, and 330I-341C loop. For DsKabC-PKA, the flexible regions included the 32W-44A loop, 86A-130D loop, 204Y-218R loop, and 282H-344Y loop. For GfKabC-PKA, the flexible regions included the 95T-123D loops, 129F-148H loops, 180A-213L loops, and 313T-325Y loops (Figure 9). The conformational flexibility of the loops around the active center provided the necessary microenvironment for catalysis. The embedding area of the substrate in kainoid synthases reflected the size of the binding interface between the substrate and kainoid synthases, and the binding state of the substrate and kainoid synthases could be determined by analyzing the embedding area. The buried solvent accessibility surface area (Buried SASA) reflected the size of the binding interface between the substrates and kainoid synthases. According to the analysis, the cNGG in PnDabC and the PKA in DsKabC remained stable, indicating that the contact area between cNGG and PnDabC and between PKA and DsKabC remained stable; that is, the combinations remained stable. For GfKabC-PKA, the buried SASA was relatively stable during 0–350 ns but fluctuated sharply during 350–500 ns and decreased to 0, indicating that PKA was stable when combined with GfKabC in the first 350 ns. After 350 ns, the contact area decreased, and the combination was no longer stable (Figure 10).

### 2.4. Key Structural Moieties of the Substrate Recognized by Kainoid Synthases

**Figure 11:**
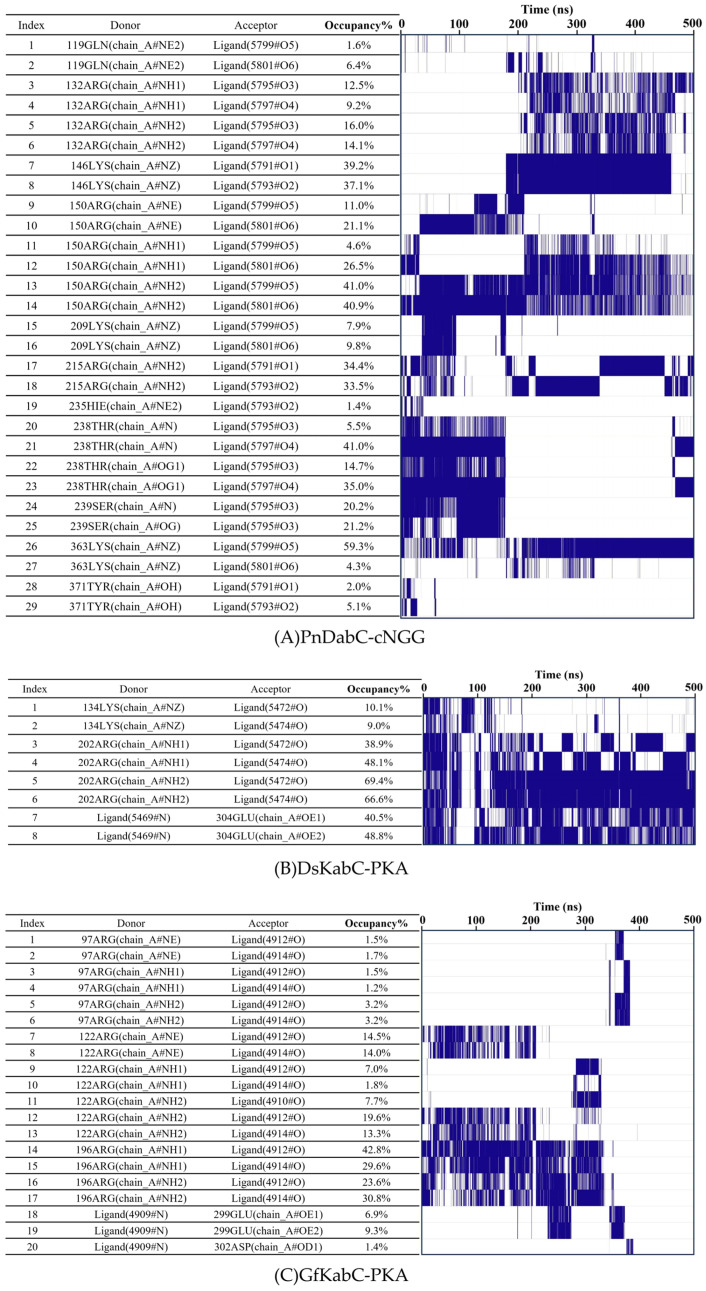
Hydrogen bond occupancy between substrates and kainoid synthases.

In order to determine the key structural part of the substrates recognized by kainoid synthases, we counted the number and frequency of hydrogen bonds between the individual functional groups of kainoid synthases and substrates in 500 ns (Figure 11). For PnDabC, we found three carboxyl groups of cNGG, namely cNGG-1COOH, cNGG-2COOH, and cNGG-3COOH, were always the key sites for recognition, the cNGG-3COOH from the geranyl pyrophosphate formed the most hydrogen bonds with the PnDabC. In total, 29 hydrogen bonds were formed between PnDabC and cNGG; among them, 12 hydrogen bonds were accepted by the cNGG-3COOH from geranyl pyrophosphate, for which the donors were 119Q, 150R, 209K and 363K, respectively. The two carboxyl groups, cNGG-1COOH and cNGG-2COOH, from L-Glu accepted 22 hydrogen bonds donated by 146K, 215R, 371Y, 132R, 238T, 239S, and 235H, respectively. Among them, cNGG-1COOH accepted seven hydrogen bonds donated by 146K, 215R, and 371Y, while cNGG-2COOH accepted 10 hydrogen bonds donated by 132R, 238T, 239S, and 235H. According to the frequency of hydrogen bond formation, it can be divided into two periods; in the first 200ns, cNGG mainly accepted hydrogen bonds donated by 371Y, 235H, 238T, 239S, 150R, and 209K, indicating that it may be the binding stage of cNGG and PnDabC. The higher the occupancy, the more critical residues needed to bind. In the latter 300 ns, cNGG mainly formed hydrogen bonds with 119Q, 150R, 132R, 146K, 215R, 239S, and 363K, among which, the occupancy of cNGG-3COOH and 363K reached 59.3%, revealing that 363K is the key residue in the catalytic process of PnDabC. Compared with PnDabC, DsKabC formed fewer hydrogen bonds with PKA. PKA has two carboxyl groups, and only PKA-1COOH accepted hydrogen bonds donated by 134K and 202R.

Furthermore, the nitrogen atom of PKA donated hydrogen bonds to 304E. After 140 ns, the PKA-1COOH accepted stable hydrogen bonds donated by NH1 and NH2 of 202R, and the average occupancy rates were 43.5% and 68%, respectively. The OE1 of 304E also accepted two stable hydrogen bonds donated by nitrogen atoms of PKA. Compared with PnDabC and DsKabC, the hydrogen bond between GfKabC and PKA only existed in the first 400 ns, and only PKA-1COOH in two carboxyl groups accepted hydrogen bonds donated by 3 arginine. Furthermore, nitrogen atoms of PKA donated hydrogen bonds to 299E and 302D, respectively. In the first 200 ns, 122R and 196R donated hydrogen bonds to PKA-1COOH, respectively. The highest hydrogen bonds occupancy was 42.8%, which was lower than PnDabC and DsKabC. After 200 ns, the hydrogen bonds between PKA and GfKabC were volatile, and there were no hydrogen bonds after 400 ns. The hydrogen bond occupancy and hydrogen bond receptors of DsKabC and GfKabC showed that γ-carboxyl group of PKA was the key recognized group. For PnDabC, the three carboxyl groups of cNGG were all key recognized groups.

### 2.5. Key Residues Responsible for Interacting with the Substrates

**Figure 12:**
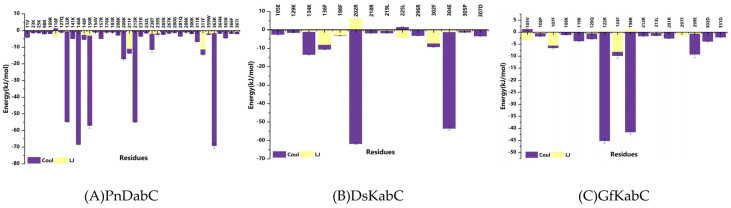
Interaction energy between the substrate and kainoid synthases.

**Figure 13:**
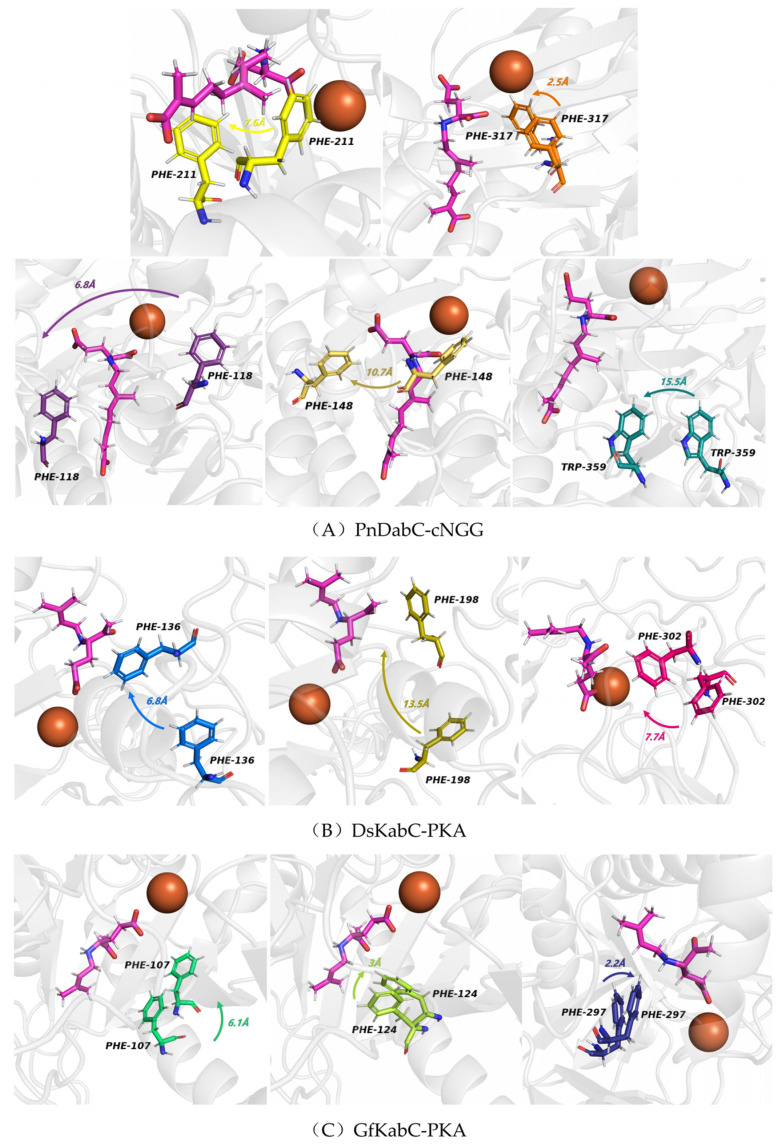
Conformational transformation of aromatic residues during a specific 50 ns window in the catalytic process (the orange ball model is Fe^2+^, the pink stick-ball models are substrates, the stick-balls in other colors are aromatic residues, the arrows and numbers indicate the direction and angle of rotation of the aromatic residues).

**Figure 14:**
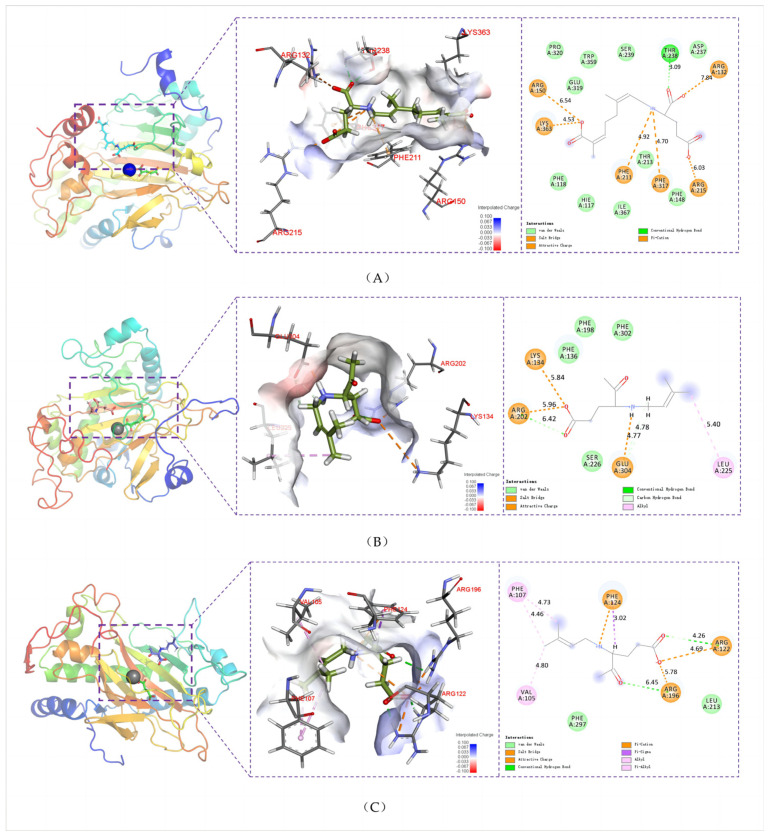
Interaction between key residues and kainoid synthases on a simulated endpoint. (**A**): PnDabC-cNGG; (**B**): DsKabC-PKA; (**C**): GfKabC-PKA.

At present, it has been widely recognized that key residues play an important role in the biosynthesis of terpenoids, such as aromatic residues stabilizing carbocation intermediates through π-cation interactions [11,12]. In order to determine the key residues that kainoid synthases bind to on the substrates, we calculated the interaction energy between the substrate and each active site residue in 500 ns simulation, and analyzed the conformational changes of the key residues. We found that electrostatic interactions played a decisive role between kainoid synthases and substrates (Figure 12). In PnDabC, the Coulomb forces of 132R, 146K, 150R, 209K, 215R, 238T, and 363K and the Lennard-Jones potentials of 211F and 317F dominated binding to cNGG. In previous studies, GfKabC showed much more catalytic promiscuity, though the reason for this was not clear [8,10]. Interestingly, we chose a 50 ns window in the catalytic process to analyze the conformational transformations of key residues, and found that after binding with cNGG, the benzene rings of 118F, 148F, 211F, 317F, and 359W rotated towards cNGG-1COOH, and the rotation of the side chains of aromatic residues would not lead to any highly feasible substrate conformational change (Figure 13). This may be the reason for less catalytic promiscuity of PnDabC, and these conformational transformations improved the interaction with cNGG. In DsKabC, the benzene rings of 136F, 198F, and 302F rotated towards PKA-1COOH, while in GfKabC, 107F, 124F, and 297F also rotated towards PKA-1COOH. In the DsKabC-PKA complex, the aromatic amino acids were closer to PKA-1COOH than in the GfKabC-PKA complex during the catalytic process. These findings suggest that residue-level conformational transitions of kainoid synthases also play a role in substrate binding and subsequent catalysis. From the analysis of the simulated endpoint kainoid synthase-substrate conformations, we found that three aromatic amino acids formed van der Waals forces with PKA in DsKabC, while the aromatic amino acids in GfKabC formed van der Waals forces and the hydrophobic interactions of π-cation and π-alkyl (Figure 14). According to the contribution analysis of residues, we found that the van der Waals forces of these residues were much stronger than the hydrophobic interactions (Table S1), so PKA in DsKabC was more stable during the catalytic process and DsKabC lacked catalytic promiscuity compared with GfKabC.

**Figure 15:**
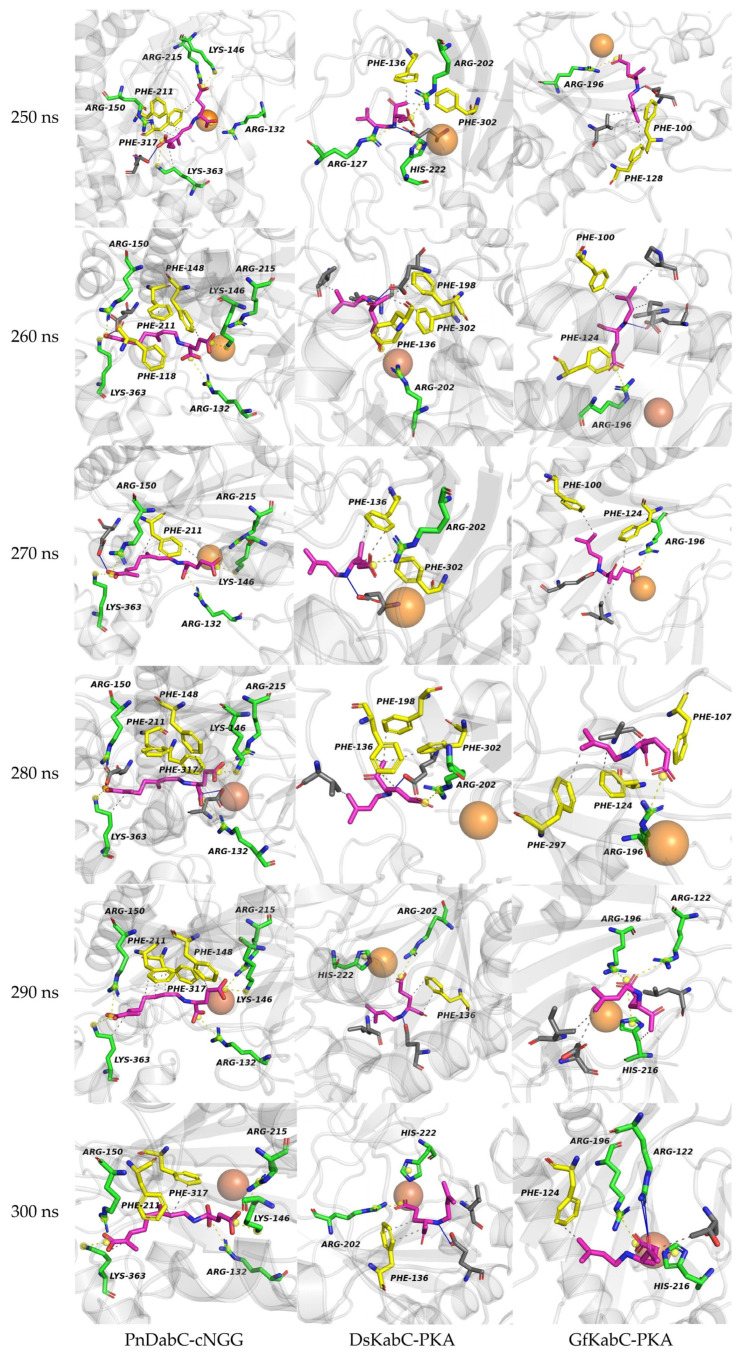
The number of aromatic residues and basic amino acids in the catalytic pocket of kainoid synthases between 250 ns and 300 ns (the orange ball model is Fe^2+^, the pink stick-ball models are substrates, the yellow stick-ball models are aromatic residues, the green stick-ball models are basic amino acid residues).

**Figure 16:**
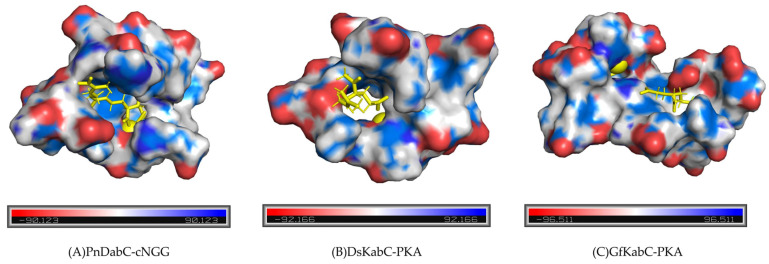
The relaxation level and electrostatic potential energy of the active pocket of kainoid synthases at the midpoint of the simulation.

We selected the conformational changes during the 250 ns to 300 ns period of the kainoid synthases catalytic process for further study (Figure 15). We found that, between 250 ns and 300 ns, PnDabC contained eithor more aromatic residues or more basic amino acids in its substrate-binding catalytic pocket compared to DsKabC and GfKabC. Aromatic residues can form π-cation interactions to stabilize substrates and intermediates, which may result in weaker catalytic promiscuity. In DsKabC, during the same period, the catalytic pocket contained eithor more aromatic residues or more basic amino acids than in GfKabC, making DsKabC more capable of forming π-cation interactions to stabilize substrates and intermediates. Conversely, the catalytic pocket of GfKabC contained the fewest aromatic residues and basic amino acids, lacking sufficient π-cation interactions to stabilize substrates and intermediates, making it less able to prevent alternative catalytic pathways and, thus, displaying stronger catalytic promiscuity. Additionally, at the midpoint of the simulation, GfKabC exhibited a more relaxed active pocket compared to PnDabC and DsKabC (Figure 16); here, we used the tool KVFinder for cavity detection and characterization of any type of biomolecular structure, with the results showing that GfKabC had a larger cavity volume than PnDabC and DsKabC (Table 1). The electrostatic potential energy of its active pocket was the lowest. This indicates that the active pocket was more flexible, facilitating intermediates to react towards other catalytic pathways.

### 2.6. Binding Energy Analysis of Kainoid Synthases

In the case of solvation energy, RMSD, Rg, distance, Buried SASA, and interaction energy were comprehensively considered to select the complex trajectory in a stable state. The MM-PBSA (Molecular Mechanics–Poisson Boltzmann Surface Area) method was used to calculate the binding-energy-related terms, as shown in the Table 2. From the enthalpy point of view alone, since the water box is a polar environment, the protein cavity is usually a non-polar environment, so the substrate is easy to bind to water, but not easy to bind to enzymes. On account of the fact that substrates cNGG and PKA are easy to dissolve in water, we considered the ΔEpol. In the PnDabC-cNGG complex, the electrostatic interaction ΔEele was higher than the van der Waals force interaction energy ΔEvdw, the former being 3.1 times the latter. Both ΔEele and ΔEvdw were much higher than the hydrophobic interaction ΔEnonpol. Therefore, in the composition of the PnDabC-cNGG binding energy, the electrostatic interaction played a major role and the van der Waals force interaction played a minor role, while the hydrophobic interaction played a complementary role. The ΔEMMPBSA of PnDabC-cNGG was 74.724 ± 6.145 kJ/mol, which was greater than zero. This is because small molecules carry more charges and have larger electrostatic interactions, which makes the polar solvation energy larger, resulting in the final calculated binding energy being greater than zero.

The electrostatic interaction ΔEele in the DsKabC-PKA complex was higher than the van der Waals force interaction energy. The former was 3.2 times the latter, and both were much higher than the hydrophobic interaction ΔEnonpol. Therefore, in the composition of DsKabC-PKA binding energy, the electrostatic interaction played a major role, the van der Waals force interaction played a secondary role, and the hydrophobic interaction played a supplementary role. The ΔEMMPBSA of DsKabC-PKA was 34.465 ± 2.689 kJ/mol, which was greater than zero due to polar solvation energy as well.

For the GfKabC-PKA complex, the electrostatic interaction ΔEele in the complex was higher than the van der Waals force interaction energy ΔEvdw, being three times ΔEvdw. Both were much higher than the hydrophobic interaction ΔEnonpol. Therefore, in the composition of GfKabC-PKA binding energy, the electrostatic interaction played a major role, the van der Waals force interaction played a secondary role, and the hydrophobic interaction played a supplementary role. The ΔEMMPBSA of GfKabC-PKA was −15.972 ± 4.005 kJ/mol. ΔEpol of GfKabC-PKA was the smallest, which indicates that more easily dissolved in the simulation process of GfKabC-PKA and the water molecule had the greatest influence on the complex system.

Enzyme promiscuity is a modern term that is not fully understood at present. Catalytic promiscuity refers to the ability of enzymes to catalyze different chemical reaction processes, through different transition states, to produce different intermediates or products. Catalytic promiscuity has attracted more attention in the field of protein engineering, as it can be used to enhance natural catalytic activity or obtain new catalytic functions. In the process of biosynthesis of natural products, enzyme-catalyzed promiscuity is not uncommon and is closely related to the chemical diversity of natural products. In the biosynthesis of terpenoids, the substrates can be transformed into various products through the cyclization, rearrangement, and quenching cascade reactions involving carbocations. Therefore, the key to studying the catalytic mechanism of terpene natural product biosynthase is to explore the root cause of catalytic promiscuity or catalytic specificity in different terpene natural product biosynthetic processes. In theoretical studies of terpenoid biosynthesis, the importance of π-interactions between aromatic residues (such as PHE/TYR/TRP) and carbocation ions has been mentioned many times [13,14,15,16,17,18,19]. The two main roles of aromatic residues in the catalysis of kainoid synthases are to restrict the orientation of substrates through steric hindrance brought about by large side chains, and to stabilize carbocation intermediates and transition states through π-cation interactions [20]. According to the analysis, PnDabC is catalytically specific compared to DsKabC and GfKabC, whereas GfKabC has been shown to be catalytically promiscuous in previous studies [8]. By analyzing the interaction between carbocation intermediates and key residues in active pockets, and the key chemical regulatory factors of terpenoid natural product biosynthase during the reaction process, the sources of chemical diversity of terpenoid compounds were revealed, enabling a better understanding and use of the catalytic promiscuity and specificity of terpenoid natural product biosynthase.

Kainoid synthases belong to Fe^2+^ and alpha-ketoglutarate-dependent dioxygenases, which are extremely versatile biocatalysts in nature. They play a key role in the biosynthesis of meroterpenoids and have been less studied, including structural characteristics, catalytic mechanisms, engineering development, and the discovery of new enzyme properties. At present, there are very few studies on meroterpenoids synthase, and those that do exist have mainly focused on the meroterpenoids synthase of terpenoids and polyketones. The current research on kainoid synthases only focuses on the catalytic mechanism or expression and purification, without analyzing the conformational dynamics and catalytic promiscuity [21,22]. The structure–activity relationship of meroterpenoids synthases from marine algae has not been fully researched. The analysis of the mechanism of kainoid synthases is expected to further elucidate the biosynthesis process of kainoids.

## 3. Materials and Methods

### 3.1. Protein Preparation and Evaluation

The amino acid sequences of PnDabC, DsKabC, and GfKabC were downloaded from NCBI (https://www.ncbi.nlm.nih.gov/, accessed 30 November 2023). The protein structures of PnDabC, DsKabC, and GfKabC were from Alphafold2 (https://alphafold.com/, accessed 22 November 2023) [23,24]. Evaluation of PnDabC, DsKabC, GfKabC protein models by SWISS-MODEL [25]. Ramachandran plots made by SWISS-MODEL, too.

### 3.2. Molecular Dynamics Simulations

All molecular dynamics simulations were performed using the GROMACS 2018.8 program. The GAFF force field was used for small molecules, the AMBER14SB force field and TIP3P water model were used for proteins, and files of proteins and small molecular ligands were combined to construct a simulation system of the complex. The molecular dynamics simulation (MD) was carried out under constant temperature and pressure and periodic boundary conditions. In the MD simulation process, all the hydrogen bonds involved were constrained by the LINCS algorithm, and the integration step was 2 fs. The electrostatic interaction was calculated using the Particle-Mesh Ewald (PME) method, and the cutoff value was set to 1.2 nm. The non-bond interaction cutoff was set to 10 Å and updated every 10 steps. The V-rescale temperature coupling method was used to control the simulated temperature at 298 K, and the Berendsen method was used to control the pressure at 1 bar. At 298 K, NVT and NPT equilibrations of 100 ps each were simulated, and MD simulations of 100 ns were performed for the complex system, preserving conformations every 10 ps. After the simulation was completed, the simulated trajectories were analyzed, and the MMPBSA binding free energy of the complex was analyzed using the g_mmpbsa program. Table S1 lists the residues contribution of these kainoid synthases. It is worth noting that the KA-lactone content of GfKabC products is higher than that of KA, while PnDabC and DsKabC are opposite. Their structural and functional features make them good objects for studying the structure–activity relationship of kainoid synthases. The complete simulation videos of the three kainoid synthases are provided in the Supplementary Information.

### 3.3. Data Analysis

GROMACS internal tools were used to analyze the biophysical properties of kainoid synthases. When the acceptor–donor distance is less than 0.35 nm and the acceptor–donor angle is less than 30°, it is defined as a gmx_hbond [26]. RMSD (gmx_rms) and RMSF (gmx_rmsf) superimpose the Cα atoms of each snapshot structure onto the initial structure by least squares fitting. After eliminating the overall translational and rotational motion, RMSD (gmx_rms), RMSF (gmx_rmsf), distance (gmx_distance), Rg (gmx_gyrate), and SASA (gmx_sasa) were measured to check the stability and flexibility of the structure [27,28,29]. The interaction energy (gmx_energy) between amino acid residues and the substrate was calculated based on the CHARMM 36 force field [30]. Structure visualization was performed using Pymol [31]. The substrate was docked to the enzyme binding site using the Autodock program. The Notepad++ v8.2.1 was used to make a docking file and the PLIP (Protein–Ligand Interaction Profiler) was used to analyze the non-covalent interactions of protein–ligand complexes at the atomic level [32].

## 4. Conclusions

In summary, through modeling, molecular docking, and molecular dynamics simulations, we discovered that kainoid synthases exhibit different conformational changes during the catalytic process. GfKabC has a lower binding energy and, compared to PnDabC and DsKabC during the simulation process, it has a more relaxed active pocket and lower electrostatic potential energy of its active pocket. The higher number of aromatic residues and basic amino acids in the catalytic pockets of PnDabC and DsKabC leads to the formation of more π-cation interactions that stabilize the intermediates. The rotation of the side chains of aromatic residues is less in GfKabC-PKA; thus, it would not prevent feasible substrate conformational change. Compared with PnDabC and DsKabC, GfKabC cannot stabilize substrates and intermediates, making it less able to prevent alternative catalytic pathways and, thus, displaying stronger catalytic promiscuity. These findings provide theoretical support for understanding the structure–activity relationship and the mechanism of kainoid synthases.

## Figures and Tables

**Table 1:** The volume cutoff (Å3) of the activity pocket in the middle point of the simulation.

Index	Area (Å2)	Vol. (Å3)	Avg Dep.(Å)	Max Dep.(Å)	Avg Hyd.
PnDabC-cNGG	680.81	376.92	1.67	4.41	−0.02
DsKabC-PKA	667.45	408.46	1.61	5.5	0.17
GfKabC-PKA	730.44	438.26	1.47	4.41	0.15

**Table 2:** Binding energy and its composition in stable state (unit: kJ/mol).

Complex	PnDabC-cNGG	DsKabC-PKA	GfKabC-PKA
ΔEvdw	−133.164 ± 6.729	−71.61 ± 1.427	−60.76 ± 5.734
ΔEele	−411.14 ± 25.248	−230.503 ± 3.59	−186.169 ± 19.89
ΔEpol	641.384 ± 24.122	351.107 ± 4.775	244.753 ± 21.658
ΔEnonpol	−22.355 ± 0.216	−14.529 ± 0.031	−13.796 ± 0.807
ΔEMMPBSA	74.724 ± 6.145	34.465 ± 2.689	−15.972 ± 4.005
−TΔS	82.94 ± 6.966	32.564 ± 2.885	53.314 ± 6.549
ΔGbind *	157.664 ± 4.835	67.029 ± 4.931	37.342 ± 2.572

* ΔGbind = ΔEvdw + ΔEele + ΔEpol + ΔEnonpol − TΔS.

## Data Availability

The data presented in this study are available on request from the corresponding author.

## Supplementary Materials

The following supporting information can be downloaded at: https://www.mdpi.com/article/10.3390/md22070326/s1, Full simulation process of PnDabC-cNGG (extracting one frame every 5 ns). Full simulation process of DsKabC-PKA (extracting one frame every 5 ns). Full simulation process of GfKabC-PKA (extracting one frame every 5 ns). Table S1. Residues contribution of kainoid synthases.
